# Validity of personality measurement in adults with anxiety disorders: psychometric properties of the Spanish NEO-FFI-R using Rasch analyses

**DOI:** 10.3389/fpsyg.2015.00465

**Published:** 2015-04-21

**Authors:** Felix Inchausti, Joe Mole, Eduardo Fonseca-Pedrero, Javier Ortuño-Sierra

**Affiliations:** ^1^Mental Health Services, University Hospital of BadajozBadajoz, Spain; ^2^Universidad de SalamancaSalamanca, Spain; ^3^Oxford UniversityOxford, UK; ^4^Universidad de La RiojaLa Rioja, Spain

**Keywords:** five factor model, personality measurement, NEO Five Factor Inventory, item response theory, Rasch model

## Abstract

The aim of this study was to analyse the psychometric properties of the Spanish NEO Five Factor Inventory–Revised (NEO-FFI-R) using Rasch analyses, in order to test its rating scale functioning, the reliability of scores, internal structure, and differential item functioning (DIF) by gender in a psychiatric sample. The NEO-FFI-R responses of 433 Spanish adults (154 males) with an anxiety disorder as primary diagnosis were analysed using the Rasch model for rating scales. Two intermediate categories of response (‘neutral’ and ‘agree’) malfunctioned in the Neuroticism and Conscientiousness scales. In addition, model reliabilities were lower than expected in Agreeableness and Neuroticism, and the item fit values indicated each scale had items that did not achieve moderate to high discrimination on its dimension, particularly in the Agreeableness scale. Concerning unidimensionality, the five NEO-FFI-R scales showed large first components of unexplained variance. Finally, DIF by gender was detected in many items. The results suggest that the scores of the Spanish NEO-FFI-R are unreliable in psychiatric samples and cannot be generalized between males and females, especially in the Openness, Conscientiousness, and Agreeableness scales. Future directions for testing and refinement should be developed before the NEO-FFI-R can be used reliably in clinical samples.

## Introduction

The Five Factor Model (FFM) of personality has become the reference taxonomy for the study of both general and clinical personality traits (Clark, 2007; Gore and Widiger, 2013). The terms commonly used to describe the personality traits that underpin the FFM are Openness, Conscientiousness, Extraversion, Agreeableness, and Neuroticism, known by the acronym OCEAN (Costa and McCrae, 1985). The FFM has several characteristics that make it a very helpful model: (1) it integrates terminology from diverse theoretical frameworks which facilitates communication between researchers, (2) it makes it easier to explore the relationship between personality and other phenomena, and (3) it is an efficient model that provides a simple outline of general personality structure (De Raad and Perugini, 2002). In recent years, numerous studies have associated the FFM domains with psychiatric disorders, particularly personality disorders (Widiger and Costa, 2002; Mullins-Sweatt and Widiger, 2006), and have provided empirical support for the value of understanding the DSM-IV (e.g., Saulsman and Page, 2004) and DSM-5 (e.g., Trull and Widiger, 2013) personality disorders in terms of the FFM traits.

Other studies have found that the FFM traits may be a key mediator in the utilization, time course, and effectiveness of various treatments of mental disorders (Hopwood et al., 2008). Furthermore, some research suggests an interaction between FFM traits and the modalities of treatment for mental disorders (Few et al., 2010). For example, Miller et al. (2006) examined the relationship between FFM traits and treatment utilization in depression, anxiety, and personality disorders. Their results showed openness to experience and conscientiousness significantly predicted the number of therapy sessions needed, and treatment satisfaction, and compliance. Moreover, they found medication use was significantly associated with low scores on extraversion and high scores on agreeableness. Although these preliminary results are limited, they support the potential utility of the FFM in treatment planning (Hopwood et al., 2008). However, the application of the model requires appropriate instruments to measure the FFM traits in psychiatric populations.

The NEO Personality Inventory–Revised (NEO-PI–R; Costa and McCrae, 1992) is one of the most frequently used questionnaires in the literature to assess the FFM domains. The results obtained with this measurement instrument have been consistent with the FFM in samples of different ages (De Fruyt et al., 2009; Spence et al., 2012) and from different cultures and countries (Rolland et al., 1998). There are two abbreviated versions of this self-report, the NEO Five Factor Inventory (NEO-FFI; Costa and McCrae, 1992) and, the more recent, NEO Five Factor Inventory–Revised (NEO-FFI–R; McCrae and Costa, 2004). Both consist of 60 items selected from the 240-item NEO-PI–R that assess the FFM traits of personality at the domain level. These brief versions are widely used in the literature because they measure the personality traits in less time and use fewer items than the NEO-PI–R (Hosie et al., 2014). Aluja et al. (2005, 2009) found that the psychometric properties of the Spanish NEO-FFI and NEO-FFI-R adaptations are equivalent to the English original in non-clinical Spanish samples but it is not yet known how well they function, at psychometric level, with Spanish psychiatric samples. Some research suggests that instruments assessing FFM traits in non-clinical samples are valuable for assessing personality in psychiatric samples but results are inconclusive (Markon et al., 2005; Samuel et al., 2010).

In summary, extensive research highlights the need for brief and psychometrically reliable and valid instruments to assess the FFM personality traits in both non-clinical and clinical populations. Psychometric models based on item response theory (IRT), such as the Rasch Model (RM; Rasch, 1960), can provide more efficient personality measures and can improve existing measurement instruments (Inchausti et al., 2014). The RM is an alternative approach to Classical Test Theory (CTT), which solves some of CTT’s methodological drawbacks (Wright and Stone, 1979). For example, it can be used to examine the coherence of items with regard to the latent trait in question, allowing the construct validity of the questionnaire to be assessed. A further advantage is that, because participants and items are measured along the same continuum, it is easy to identify which items have been endorsed by which participants (Wilson, 2005). The aim of this study was to analyse the psychometric properties of the Spanish NEO-FFI-R using Rasch analyses in order to test its rating scale functioning, the reliability of scores, internal structure, and differential item functioning (DIF) by gender in a large psychiatric sample.

## Materials and Methods

### Participants

Participants were 433 Spanish adults (154 males) with an anxiety disorder as primary diagnosis according to DSM-IV-TR criteria (American Psychiatric Association, 2000). The mean age was 36.45 (SD = 14.14) and the primary diagnosis distribution was as follows: 122 patients (29%) had a diagnosis of panic disorder, 97 (22%) had a diagnosis of social phobia, 68 (16%) had a diagnosis of obsessive-compulsive disorder, 57 (13%) had a diagnosis of generalized anxiety disorder, 46 (11%) had a diagnosis of specific phobia, and 43 (9%) had a diagnosis of posttraumatic stress disorder. These diagnoses were established using the Structured Clinical Interview for DSM-IV-TR Axis I Disorders (First et al., 2002). Participants with psychotic symptoms, substance abuse, personality disorders, and suspected intellectual disability associated with the anxiety disorder were excluded. Most participants (67%) had one or more additional diagnosis, including other anxiety, and mood disorders; 82.3% of participants had completed secondary school, 51.1% had completed high school, and 13.6% had completed university studies. The mean number of years of education was 13.96 (SD = 5.76).

### Measures

The NEO-FFI-R and a brief sociodemographic questionnaire were administered to all participants in the context of a general clinical assessment. The Spanish version of the NEO-FFI-R (McCrae and Costa, 2004) contains 60 items selected from the NEO-PI–R (Costa and McCrae, 1999) which are summed to measure personality at the superordinate level only. Each of the five personality traits is measured using a 12-item scale and each item is rated on a 5-point Likert scale, ranging from strongly disagree (SD) to strongly agree (SA). Psychometric properties of the NEO-FFI-R scores have been previously analysed using CTT in a non-clinical Spanish sample obtaining good reliability indexes and factor structures in line with the results reported using the English original (Aluja et al., 2005, 2009).

### Procedure

Participants completed the measures during general clinical assessments in the Mental Health Services of Badajoz (MHSB, Spain). Participants were informed about the research and, after signing the consent form, were asked to complete anonymous questionnaires. They received no type of incentive for taking part in the study. The measurement instruments were always completed under the supervision of a researcher. This study was approved by the Research and Ethics Committees at MHSB.

### Data Analyses

Rasch analysis is a specific approach to construct modeling within the IRT framework. The RM provides a way of relating item difficulty and respondent characteristics. When applied to the measurement of psychological constructs, terms such as an ‘item scale value’ and an ‘individual’s attitude toward something’ can be represented in item and respondent locations. In the measurement of personality using the NEO-FFI-R, item scale value relates to the likelihood of a particular trait being endorsed, and an individual’s attitude refers to the amount that a person endorses a personality trait; the RM placed both of these on the same latent continuum.

The assumptions of the RM are different to those of many psychometric methods. For example, the model assumes that the way in which people respond is probabilistic. Thus, more questions on the neuroticism dimension of the NEO-FFI-R will be endorsed by a person with a higher level of neuroticism than a person with a lower level neuroticism, and indicators commonly reported are more likely to be endorsed than items that are rarely reported. Furthermore, it is assumed that the metric of the underlying construct is reflected by the fact that people respond to categories in an ordered manner. For each item on the NEO-FFI-R, indicator severity influences the probability of a person endorsing a response category that is high on the scale. Individual differences within the sample, such as the gender of the responder, should not affect the probability of endorsing a question (DIF). If the responses to a questionnaire meet the assumptions of the RM, it can be determined that the questionnaire has good construct validity and functions as a true interval-level measure of a latent variable.

The Rasch analyses were performed for the five NEO-FFI-R scales, using the software Winsteps (Linacre and Wright, 2000; Linacre, 2013). First, the quality of the response categories were tested with the Rating Scale Model (RSM; Andrich, 2013), an extension of the RM for polytomous items. This model gives an interpretation of category ordering in rating formats, by inferring a space of experimentally independent Bernoulli variables, characterized by Rasch’s simple logistic model, from a complex of mathematical relationships among response spaces (Andrich, 2013). Linacre (2002) has proposed several criteria for diagnosing a malfunctioning empirical rating scale. A scale can be considered to be performing at an optimal level when: (a) all the categories are used frequently to estimate step calibrations, or when there are unimodal or bimodal distributions with the highest frequencies in the extreme categories. (b) The average person measures by category move up the rating scale monotonically. (c) The fit of persons, items, and categories can be assessed using averaged residuals. The degree of fit is indicated by the statistics *Outfit*, the averaged standard squared residuals, and *Infit*, the averaged standard squared residuals, weighted by the information function. For both statistics, the expected value is 1. Values higher than this point to patterns that are abnormal with respect to the model and values lower than this indicate overfit, i.e., response patterns that are deterministic. When empirical data are not predictable from the model, values will be substantially higher than 1. Linacre (2002) also states that category misfit is indicated by *Outfit* values of more than 2. (d) Within the variable there should be an instance in which the probability of responding in a category is higher than the probability of choosing any other category, i.e., within the adjacent categories the step calibrations must advance monotonically. It is also recommended that step difficulties should advance by at least 1.4 *logits* and by no more than 5.0 *logits*.

After testing the rating scale, the fit of the data to the RM was analysed in the five NEO-FFI-R scales. The assumption of unidimensionality was examined using Principals Components Analysis of Rasch measures and residuals. It can be affirmed that the data are essentially one-dimensional if the Rasch measurement shows a moderately high percentage of explained variance (at least 20%) and the first residuals components of the unexplained variance are less than 2. Finally, DIF by gender analyses were conducted in order to probe the generalized validity. DIF was considered to be present if there were significant differences of more than a half *logit* between the difficulty parameters in males and females. An item presents DIF when the probability of a score in individuals with the same level in the latent trait varies according to the group to which they belong (e.g., gender). The standardized localization parameter differences by gender were calculated after possible sample-related differences in the distribution of the NEO-FFI-R scales were adjusted for and a Bonferroni multiple-comparison correction of the chosen significance level was made (Linacre, 2013).

## Results

### Rating Scale Functioning

**FIGURE 1:**
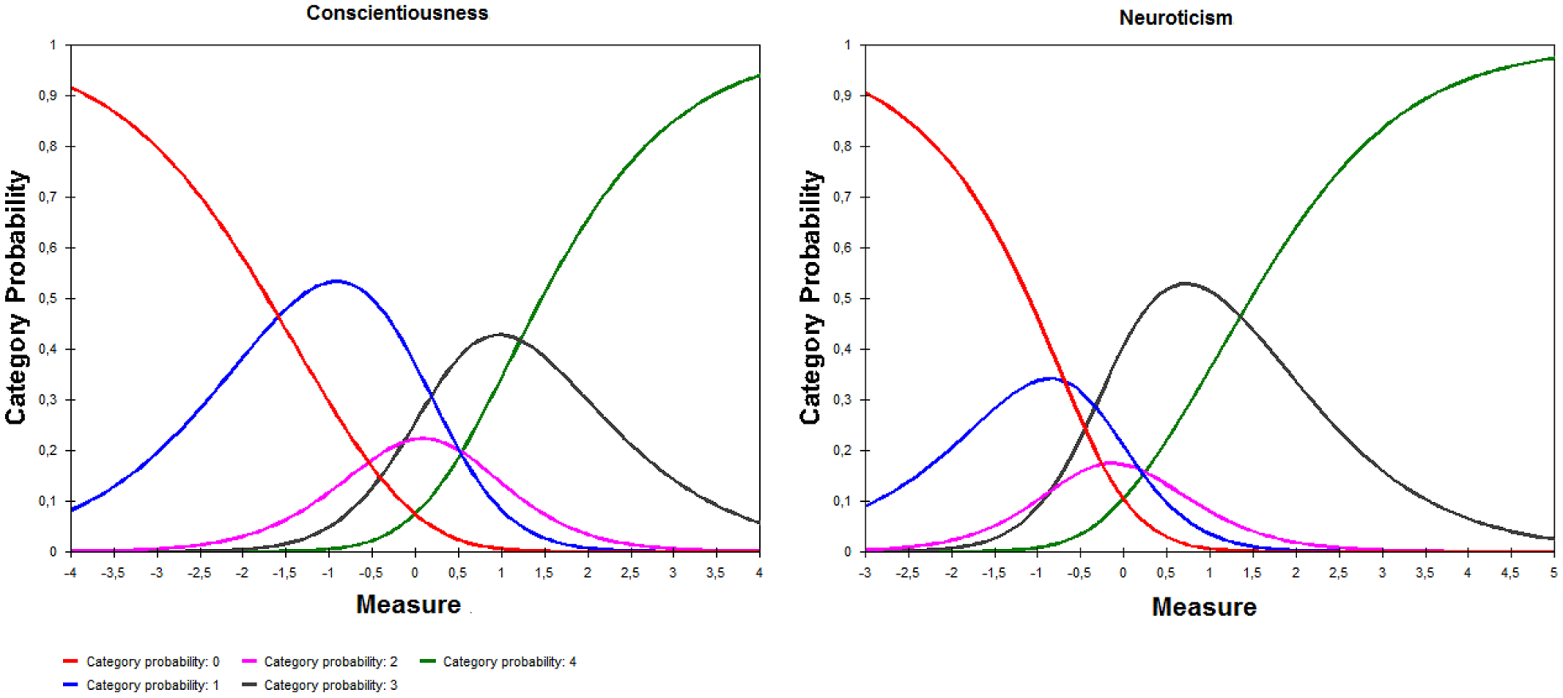
**Category curves in the Conscientiousness and Neuroticism scales**.

Testing of the quality of the response categories with the RSM indicated that the category thresholds were disordered in the Neuroticism and Conscientiousness scales (see **Table 1**). In addition, the average person measures by category advanced monotonically with the rating scale but the step calibrations did not advance monotonically with the categories in the Conscientiousness scale. The average person measures and the step calibrations were both disordered in the Neuroticism scale. Thus, in both NEO-FFI-R scales, there was no interval wherein the probability of being observed (or responding) in some of the categories was higher than the probability of choosing any other one. **Figure 1** clearly shows that the two intermediate categories (‘neutral’ and ‘agree’) malfunctioned in both scales.

**Table 1:** Rating Scale Model category statistics for the total sample.

Scale	Category	Chosen *f* (*p*)	Average B	*Infit*	*Outfit*	Step
Openness	0 (SD) 1 (D) 2 (N) 3 (A) 4 (SA)	677 (16) 1051 (25) 1102 (27) 1056 (25) 366 (6)	-1.48 -0.93 -0.24 0.54 1.03	0.89 1.01 0.98 0.86 1.44	0.90 1.05 0.97 0.84 1.29	None -1.61 -0.67 0.09 2.19
Conscientiousness	0 (SD) 1 (D) 2 (N) 3 (A) 4 (SA)	624 (15) 1438 (35) 672 (16) 918 (22) 580 (13)	-0.95 -0.50 0.02 0.40 0.94	1.06 1.01 0.96 0.94 1.07	1.02 0.97 1.15 0.85 1.06	None -1.59 0.51 -0.14 1.22
Extraversion	0 (SD) 1 (D) 2 (N) 3 (A) 4 (SA)	1321 (32) 1127 (27) 725 (17) 735 (18) 344 (6)	-2.63 -1.18 -0.29 0.56 1.07	1.06 1.02 0.88 0.96 1.29	1.17 0.88 0.78 1.02 1.29	None -1.73 -0.34 0.12 2.00
Agreeableness	0 (SD) 1 (D) 2 (N) 3 (A) 4 (SA)	304 (5) 726 (17) 1093 (26) 1336 (32) 793 (19)	-0.40 -0.07 0.18 0.73 1.54	1.01 1.22 1.01 0.83 0.82	1.07 1.70 1.04 0.82 0.87	None -1.54 -0.38 0.28 1.64
Neuroticism	0 (SD) 1 (D) 2 (N) 3 (A) 4 (SA)	146 (4) 287 (7) 340 (8) 1837 (42) 1742 (40)	0.15 0.10 0.57 0.92 1.71	1.40 0.95 1.02 1.06 0.90	1.71 0.96 0.95 0.93 0.95	None -0.69 0.19 -0.86 1.35

*SD, strongly disagree; D, disagree; N, neutral; A, agree; SA, strongly agree; Average B is the mean estimated ability in that category*.

### Psychometric Properties of the NEO-FFI-R

Model reliabilities were 0.82 in Openness, 0.83 in Conscientiousness, 0.90 in Extraversion, 0.55 in Agreeableness, and 0.69 in Neuroticism, and raw score reliabilities (Cronbach *alpha*) were 0.81 in Openness and Conscientiousness, 0.92 in Extraversion, 0.50 in Agreeableness, and 0.64 in Neuroticism. Item fit indexes, difficulty, SE of the difficulty estimate, and point-biserial item-dimension correlation of all NEO-FFI-R items are presented in **Table 2**. The item fit values indicated each scale had items that did not achieve moderate to high discrimination on its dimension (*n* = 7), particularly in the Agreeableness scale (*n* = 3). It should be highlighted, these items with RM misfit also had minor correlations with the total score for their scales in *logits*, especially in two items from Agreeableness (*r*_id_ < 0.10). Furthermore, the item relating to ‘manipulation’ showed a negative correlation with its dimension (*r*_id_ = -0.08), which indicates that it was not in the same polarity as the scale (Linacre and Wright, 2000).

**Table 2:** Item fit indexes, item difficulty (*D*_i_), SE of the difficulty estimate (SE), and correlation item-dimension (*r*_id_) for the NEO-Five Factor Inventory–Revised (NEO-FFI-R) items.

Item and content	*D*	SE	*r*	*Infit*	*Outfit*
O1 Wave of excitement	-0.06	0.06	0.80	0.45	0.45
O2 Poetry (R)	-0.05	0.06	0.57	1.00	0.98
O3 Curiosity	-0.35	0.06	0.57	0.82	0.84
O4 Patterns	0.26	0.06	0.75	0.79	0.79
O5 Controversial speakers (R)	0.18	0.06	0.48	0.77	0.84
**O6 Daydreaming (R)**	**-0.37**	**0.06**	**0.32**	1.96*	1.84*
O7 Interest in speculating	0.39	0.06	0.62	0.86	0.88
O8 Human condition	0.45	0.06	0.76	0.65	0.65
O9 Enjoy theories	0.99	0.07	0.65	0.77	0.78
O10 Emotional experiences	-1.72	0.07	0.46	1.12	1.10
O11 New hobbies	1.19	0.07	0.54	1.31	1.21
O12 Notice moods (R)	-0.91	0.06	0.40	1.47	1.37
C1 Organized (R)	0.74	0.06	0.57	0.97	0.85
C2 Clear goals	0.70	0.06	0.69	0.79	0.79
C3 Accomplish goals	-0.37	0.05	0.61	0.59	0.69
C4 Productive	0.42	0.06	0.68	1.16	1.03
C5 Neat	-0.64	0.05	0.48	1.16	1.05
C6 Perform thoroughly	-0.54	0.05	0.61	0.73	0.70
C7 Strive for excellence	-0.19	0.05	0.59	1.26	1.24
C8 Reliable	-0.09	0.05	0.66	0.88	0.84
C9 Counted on	-0.16	0.05	0.57	0.55	0.96
**C10 Methodological (R)**	**-0.44**	**0.05**	**0.26**	2.16**	2.12**
C11 Pace myself (R)	0.89	0.07	0.62	0.67	0.59
C12 Wasted time (R)	-0.32	0.05	0.54	1.11	1.10
E1 Cheerful	-0.70	0.07	0.76	0.69	0.83
E2 Enjoy talking	-0.86	0.07	0.85	0.82	0.82
E3 Fast-paced	0.80	0.07	0.70	1.11	0.91
E4 Bursting with energy	-0.59	0.07	0.76	0.78	0.79
E5 People around	0.66	0.07	0.73	0.75	0.99
E6 Do things alone (R)	0.52	0.07	0.67	1.38	1.04
E7 Active	-0.55	0.07	0.62	1.36	1.44
E8 Leader of others (R)	0.82	0.08	0.64	1.12	0.89
E9 Crowds (R)	1.31	0.08	0.67	0.91	0.77
**E10 Laugh easily**	**-1.08**	**0.07**	**0.55**	1.82*	1.99*
E11 Like action	0.57	0.07	0.77	0.57	0.55
E12 ‘Light hearted’ (R)	-0.90	0.07	0.83	1.02	1.05
A1People like me	0.54	0.06	0.63	0.99	0.97
**A2 Take advantage (R)**	**-1.14**	**0.07**	**0.10**	1.69*	1.68*
**A3 Manipulation (R)**	**-1.58**	**0.08**	**-0.08**	2.02**	2.32**
**A4 Arguments (R)**	**-0.86**	**0.07**	**0.12**	1.88*	1.98*
A5 Co-operate	0.60	0.06	0.58	1.24	1.24
A6 Thoughtful	0.22	0.06	0.47	0.73	0.74
A7 Calculating (R)	0.34	0.06	0.49	0.53	0.53
A8 Don’t like people (R)	0.90	0.06	0.58	0.74	0.75
A9 Narcissist (R)	-0.37	0.06	0.29	0.65	0.71
A10 Respectful	0.72	0.06	0.50	0.64	0.63
A11 Hot-headed (R)	0.72	0.06	0.46	0.86	0.86
A12 Egotistical (R)	-0.08	0.06	0.46	0.75	0.74
N1 Ashamed	0.52	0.06	0.37	1.06	1.35
N2 Restless (R)	-0.50	0.08	0.57	0.90	0.79
**N3 Helpless**	**0.36**	**0.06**	**0.29**	1.72*	1.95*
N4 Feel inferior	-0.27	0.07	0.61	1.17	0.91
N5 Discouraged	-0.16	0.07	0.59	0.45	0.55
N6 Go to pieces	-1.01	0.10	0.34	0.70	0.72
N7 Tense	-0.89	0.09	0.52	0.76	0.68
N8 Embittered	-0.13	0.07	0.59	0.42	0.44
N9 Worrier (R)	0.24	0.06	0.35	0.91	1.12
N10 Sad (R)	0.16	0.06	0.37	1.15	1.48
N11 Angry	1.11	0.05	0.42	1.40	1.60^∗^
N12 Lonely (R)	0.58	0.05	0.33	1.06	1.05

*O, Openness; C, Conscientiousness; E, Extraversion; A, Agreeableness; N, Neuroticism; moderate misfit; severe misfit. Boldface indicates imbalanced with the model*.

Person fit was assessed following similar criteria. The mean and SD for fit statistics were 1.00 and 0.46 (*Infit*), and 0.98 and 0.45 (*Outfit*) in Openness, 1.03 and 0.51 (*Infit*), and 1.00 and 0.50 (*Outfit*) in Conscientiousness, 1.04 and 0.71 (*Infit*), and 1.01 and 0.73 (*Outfit*) in Extraversion, 0.97 and 0.52 (*Infit*), and 1.10 and 0.76 (*Outfit*) in Agreeableness, and finally 1.16 and 0.63 (*Infit*), and 1.05 and 0.54 (*Outfit*) in Neuroticism. For each scale, the proportion of people with *Infit* and/or *Outfit* over 2 was low (0.03, 0.6, 0.10, 0.5, 0.11, respectively).

Concerning unidimensionality, the proportion of empirical variance explained by the Rash measures was lower than expected: 0.48 in Openness, 0.42 in Conscientiousness, 0.61 in Extraversion, 0.40 in Agreeableness, and 0.32 in Neuroticism; and their variance component scree plots showed large components of unexplained variance with values for the first components higher than 2 (2.6, 2.8, 2.7, 3.5, and 2.0, respectively). These results reveal that the unidimensionality assumption was not met in any of the NEO-FFI-R scales and suggest that several latent variables were present.

**FIGURE 2:**
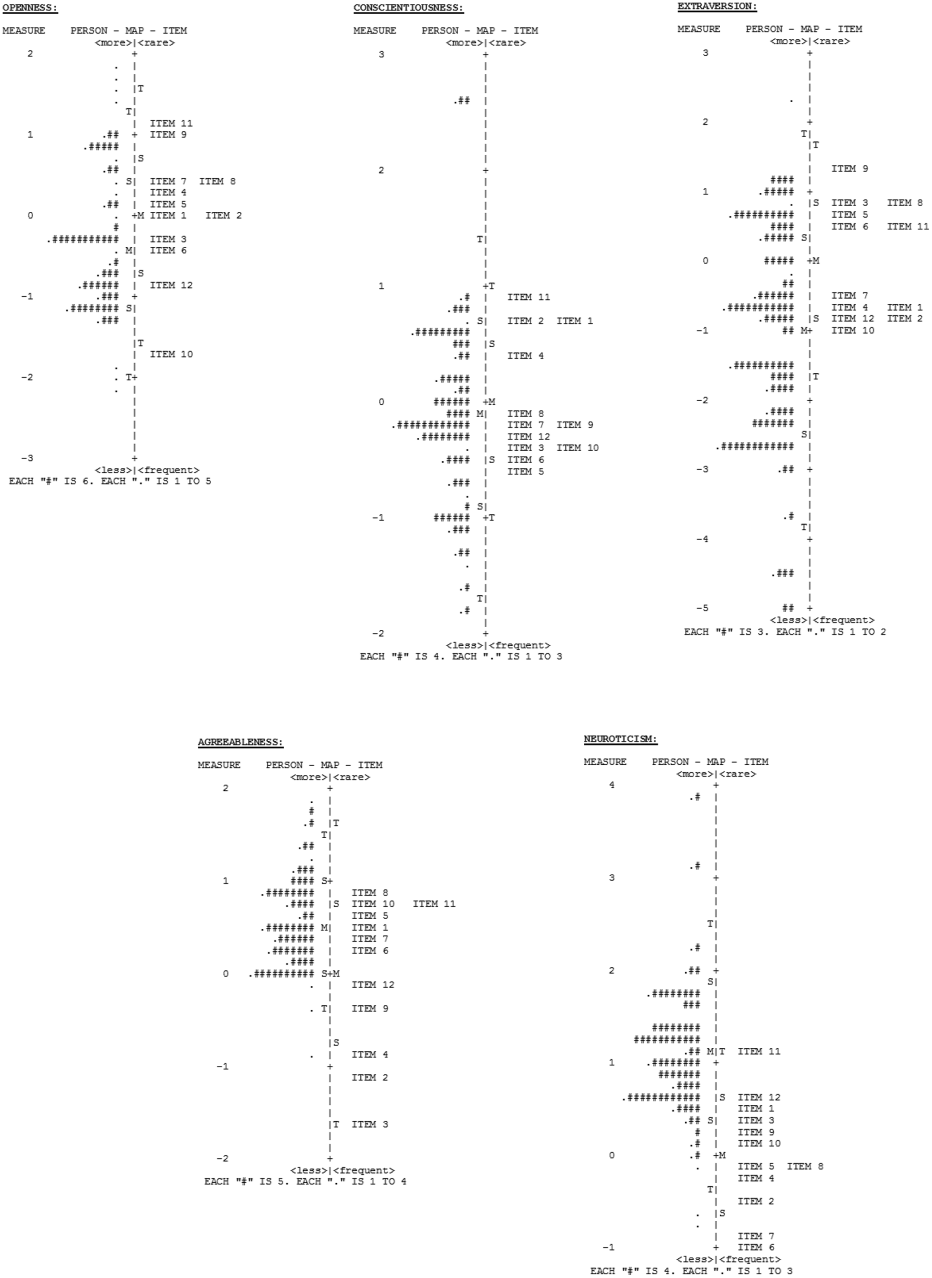
Wright maps: joint person and item representation for the five NEO-Five Factor Inventory–Revised (NEO-FFI-R) scales. Mean (*M*), 1 SD (*S*), and 2 SDs (*T*).

Wright maps, showing the conjoint representation of participants and items along the personality variables, can be seen in **Figure 2**. These maps display person ability and item difficulty estimates along interval level scales, so that the units between items, between participants and between participants and items can be read in terms of the represented variable (i.e., the five traits of personality in this case). Person and item mean (*M*), 1 SD (*S*) and 2 SDs (*T*) are located at the left and right sides of the axis, respectively, in order to facilitate the interpretation of the maps. As can be seen in **Figure 2**, participants’ levels of Neuroticism and Agreeableness were much higher than the difficulty of the items. By contrast, levels of Extroversion within the sample were below the value of the items. These results were expected, due to the characteristics of the patients and indicate the need to include items more suited to clinical populations.

### Analysis of DIF by Gender

After testing the fit of the data to the RM, DIF analyses were conducted to investigate the external validity of the NEO-FFI-R for measuring participants of different gender. DIF by gender was detected in many items (*n*= 17) from the five scales, violating the criteria proposed by Linacre (2013). A data summary of these analyses is presented in **Table 3**. As can be seen in **Table 3**, the scales which reflected less DIF by gender were Extraversion (*n* = 1) and Neuroticism (*n* = 3). Positive DIF contrast values indicate that the item was more difficult for females (*n*= 11), and negative DIF contrast values imply that the item was more difficult for males (*n* = 6).

**Table 3:** Summary of differential item functioning (DIF) analysis by gender in the NEO-FFI-R items.

Item and content	DIF		DIF contrast	*t*	*p*
	Female	Male			
O5 Controversial speakers (R)	0.41	-0.22	0.63	5.01	0.0000
O7 Interest in speculating	0.14	0.89	-0.75	-5.63	0.0000
O8 Human condition	0.64	0.12	0.52	4.09	0.0001
O9 Enjoy theories	1.22	0.61	0.61	4.52	0.0000
C1 Organized (R)	0.98	0.39	0.58	4.56	0.0000
C5 Neat	-0.38	-1.23	0.85	6.75	0.0000
C9 Counted on	-0.34	0.19	-0.52	-4.62	0.0000
C10 Methodological (R)	-0.20	-0.92	0.72	6.02	0.0000
C12 Wasted time (R)	-0.56	0.15	-0.71	-6.31	0.0000
E10 Laugh easily	-0.89	-1.45	0.56	3.98	0.0001
A2 Take advantage (R)	-1.37	-0.81	-0.56	-3.72	0.0002
A4 Arguments (R)	-0.49	-1.73	1.24	7.50	0.0000
A8 Don’t like people (R)	1.07	0.59	0.48	4.01	0.0001
A11 Hot-headed (R)	0.44	1.21	-0.76	-6.19	0.0000
N2 Restless (R)	-0.26	-1.05	0.79	4.17	0.0000
N3 Helpless	0.61	-0.23	0.84	6.06	0.0000
N11 Angry	0.68	1.82	-1.13	-10.4	0.0000

*O, Openness; C, Conscientiousness; E, Extraversion; A, Agreeableness; N, Neuroticism; DIF, differential item functioning; *t*, Rasch-Welch’s *t* contrast; *p,* probability*.

## Discussion

The testing of the NEO-FFI-R scales with the RM showed firstly that its response categories did not work appropriately on the Conscientiousness and Neuroticism scales; ‘neutral’ and ‘agree’ categories did not work well in both scales. These results suggest it would be advisable to recalibrate the rating scale for use with clinical populations. Similarly, Spence et al. (2012) found that extreme categoris (i.e., ‘strongly agree’ and ‘strongly disagree’) are generally the most commonly used NEO-FFI responses in adolescents. This suggests that intermediate categories may have only a limited utility. Thus, the rating scale is not used in the same way by all participants and this may also have played a role in the lack of unidimensionality obtained in this study, since response styles may act as a second latent variable which, in addition to trait, influence item responses (Austin et al., 2006). It is also important to note that Wetzel and Carstensen (2014) have recently shown that disordered thresholds do not impair trait measurement using the Partial Credit Model. In this regard, whether thresholds and categories are ordered with respect to the average trait estimates, are two different aspects. As can be seen for Conscientiousness in **Table 1**, the average trait estimates per category can still be ordered despite disordered thresholds. By contrast, for the Neuroticism scale the average trait estimates per category did not increase monotonically, indicating a major violation of model assumptions. A possible solution to recalibrate the rating scale in the Neuroticism and Agreeableness scales could be to combine both malfunctioned adjacent response categories into one. However, even though disordered thresholds may be caused by several different factors, including response styles, they are likely to be caused by a failure of the hypothesis behind the items (Linacre, 2002).

Secondly, the scores on the Agreeableness and Neuroticism scales were not sufficiently reliable. An explanation for these results could be the lack of items tailored to the characteristics of the sample. For example, Samuel et al. (2010) found that the items of the Neuroticism scale provided more psychometric information at the lower levels of the latent trait, and were poorer discriminators of people with high levels of Neuroticism. The current study suggests that the inclusion of more “difficult” items in the Neuroticism scale and “easier” items in the Agreeableness scale may improve their reliability indexes.

Furthermore, the fact that no NEO-FFI-R scale satisfied the unidimensionality assumption indicates that there were unknown variables that had not been taken into account, and that these variables interfered with the measurement. Once again, it was the Neuroticism and Agreeableness scales that performed particularly poorly, obtaining the worst dimensionality values. It may be that many of the trait indicators failed to discriminate the latent traits because the items were not referencing thoughts or behaviors that are relevant to people with anxiety disorders (Karsten et al., 2012). Another explanation could be found in the difficulties with item comprehension (e.g., McCrae et al., 2005).

Thirdly, the analysis of internal structure detected misfit according to the model in several items. These items also correlated weakly with the personality constructs that they were designed to asses. In the case of the Agreeableness scale, one item negatively correlated with its dimension. It is quite possible that these results were a consequence of social desirability, which could explain the poor psychometric values for the item ‘manipulation.’ Indeed, convergence between the Agreeableness scale and social desirability measures have been described in adults (Stöber, 2001).

Lastly, the DIF analysis revealed that gender influenced the difficulty of many items. The analysis suggests the NEO-FFI-R scores cannot be generalized between males and females, especially in the Openness, Conscientiousness, and Agreeableness scales. That is, the items of these scales were not measurement invariant for men and women. In addition, the direction of DIF was not balanced (11 items favored men and only six items favored women). Since DIF for gender may be explained by multidimensionality, other variables such as response style (Reise et al., 2001), educational level or psychopathological symptoms (e.g., Samuelsen, 2008) should be considered in future studies exploring the DIF by gender. On the other hand, cross-cultural research has revealed significant gender differences in the responses to the NEO-PI–R, i.e., females often report higher Neuroticism, Agreeableness, warmth (Extraversion) and openness to feelings, and males frequently report higher assertiveness (Extraversion) and openness to ideas (Costa et al., 2001). Hence, these gender differences should be taken into consideration when the measuring instruments are constructed, and the items with measurement bias by gender should be removed.

The results found in the present study should be interpreted in light of the following limitations. All participants in the study had a primary diagnosis of an anxiety disorder. As generalizability from one psychiatric population to another is often limited, the degree to which our sample is representative of other psychiatric populations is unknown. The sample size of this study did not permit the analysis of DIF according to diagnosis. A further limitation is that only one measurement instrument was used and it was not possible to control for clinical variables such as levels of anxiety, mood, or treatment effects. Although previous evidence has shown that instruments assessing FFM personality traits in non-clinical samples could be valuable for assessing personality in psychiatric samples (e.g., Markon et al., 2005), the results of the current study suggest that their scores are unreliable in a sample of adults with anxiety disorders. It should also be noted that other studies have reported problematic psychometric properties for NEO family tests in samples from the general population (Reise et al., 2001), indicating that the results of this study may not be specific to clinical samples but may indicate more general problems with the measuring instrument. In conclusion, a complete review of the NEO-FFI-R should be performed before it can be used reliably in clinical contexts, especially with the Neuroticism and Agreeableness scales, which may need more development and testing.
